# A Comparative Study of Aortic Valve Neocuspidization Techniques: Formula vs. Template Methods of Neocusp Formation

**DOI:** 10.7759/cureus.73300

**Published:** 2024-11-08

**Authors:** Roman Komarov, Abubakar I. Sidik, Maxim I Tkachev, Maxim L Khavandeev, Vladislav Dontsov, Grigorii A Esion, Ivan G Karpenko

**Affiliations:** 1 Cardiovascular Surgery, I.M. Sechenov First Moscow State Medical University, Moscow, RUS; 2 Cardiothoracic Surgery, Rossiiskii Universitet Druzhby Narodov (RUDN) University, Moscow, RUS; 3 Cardiothoracic Surgery, V.K. Gusak Institute of Emergency and Reconstructive Surgery, Donetsk, RUS; 4 Cardiothoracic Surgery, Moscow Regional Research and Clinical Institute, Moscow, RUS; 5 Cardiothoracic Surgery, A.A. Vishnevskiy Hospital, Moscow, RUS

**Keywords:** aorta, aorta surgery, aortic valve neocuspidization, aortic valve repair, aortic valve replacement, autologous pericardium, dissection, neocusps, neoleaflets, ozaki procedure

## Abstract

Introduction: The template method (TM), pioneered by Ozaki for aortic valve neocuspidization (AVNeo), has been widely adopted for aortic valve replacement, though it requires specialized instruments. This study introduces a novel formula method (FM), which uses the diameter of the aortic valve fibrous ring (AV-D) to determine the dimensions of the neocusps to be trimmed from autologous without the need for templates, potentially reducing costs and complexity. We aimed to compare the clinical outcomes of the FM with the established TM in patients undergoing AVNeo.

Methods: A retrospective and prospective study was conducted on 31 patients who underwent isolated AVNeo between January 21, 2019 and December 15, 2022. Patients were divided into two groups: FM (n = 17) and TM (n = 14). The formula for the cusp free margin horizontal length is L1 = AV-D + 10 mm, cusp height is H = AV-D, cusp suture margin is L2 is a parabola that joins L1 and H, and cusp wings to be secured to aortic sinus = 3mm. The primary endpoints were major adverse valve-related events, including cardiac death, reoperation, and infective endocarditis. Secondary endpoints included significant aortic regurgitation, peak pressure gradients, aortic valve area, and New York Heart Association (NYHA) functional class. Intraoperative times, early postoperative outcomes, and mid-term hemodynamic performance were evaluated for both techniques.

Results: Both the FM and TM demonstrated comparable intraoperative and postoperative outcomes. The cardiopulmonary bypass time, myocardial ischemia time, and blood loss were similar between the groups. Mid-term outcomes also showed no significant differences in valve function or hemodynamic parameters, with both groups exhibiting substantial reverse left ventricular remodeling. The FM group had a peak pressure gradient of 14.1 ± 4.3 mmHg compared to 18.4 ± 12.0 mmHg in the TM group (p = 0.219). The aortic valve area was 2.43 ± 0.3 cm² in the FM and 2.4 ± 0.2 cm² in the TM (p = 0.890). No significant differences were observed in freedom from reoperation or adverse events.

Conclusion: Both techniques showed excellent mid-term hemodynamic performance and comparable intraoperative and postoperative outcomes. The FM for AVNeo provides a cost-effective and practical alternative to the TM, offering similar clinical outcomes without the need for expensive templates; it has the potential to improve the accessibility of AVNeo, particularly in resource-limited settings. However, further research with larger cohorts and long-term follow-up is needed to fully assess the durability and long-term benefits of the FM.

## Introduction

The standard treatment for aortic valve disease is the implantation of either mechanical or biological valves [1]. Mechanical valves come with strict requirements, such as lifelong anticoagulant therapy and regular monitoring of the international normalized ratio. Non-compliance with these measures can result in bleeding or thrombotic complications [2,3]. Anticoagulant use also poses risks, such as teratogenic effects for young women who require surgery for aortic valve disease but wish to conceive, and adverse side effects in dialysis patients [4,5]. Moreover, mechanical valves are unsuitable for children or individuals with a narrow fibrous ring [6]. Nevertheless, mechanical prostheses tend to last longer than biological ones [7].

Biological valves, while addressing several concerns associated with mechanical valves, have their own limitations, including rapid degeneration and calcification, which eventually require reoperation. However, biological valves are less likely to result in "prosthesis-patient mismatch," where the effective orifice area is inadequate relative to the patient’s body surface area [8].

To mitigate the shortcomings of prosthetic valves, an alternative method was introduced which uses the autologous pericardium for aortic valve replacement (aortic valve neocuspidization, AVNeo) [9,10]. Although early results indicated significant degeneration and calcification of the pericardial leaflets, treating the pericardium with 0.6% glutaraldehyde has been shown to reduce the risk of leaflet retraction and scarring [11].

At present, there are numerous techniques for forming neocusps from autologous pericardium. However, many of these methods are subjective and heavily dependent on the surgeon's skill, which could affect the longevity of the neocusps [12]. Duran et al. outlined a method for complete AVNeo using autologous tissue, with a 47% freedom-from-reoperation rate over 16 years [13-15]. More recently, promising results were reported by Ozaki et al. [16,17] and Takahashi et al. [18] for AVNeo procedures that involved complete removal of the native valve.

Despite the range of available techniques for AVNeo, the most widely adopted method is neocuspidization using templates, as pioneered by Ozaki [16]. In 2007, the Japanese surgeon introduced specialized templates (JOMDD, Tokyo, Japan) whose shapes are determined by the intercommissural distance. While this method employs trimmed, asymmetrical neocusps, which may seem intuitive, long-term outcomes have shown inefficiency due to high regurgitation rates [19] and valve dysfunction [20]. Additionally, the use of commercially available templates is another drawback of this approach.

There are multiple modifications of the Ozaki procedure, with variations in the measuring devices and templates used by different authors. As previously noted, most of these techniques require additional instruments, which can complicate the reconstruction process. Another limitation of these techniques is the extended myocardial ischemia and cardiopulmonary bypass times, as the intercommissural distances are measured and the neocusps are formed only after the aortotomy, somewhat compromising the effectiveness of the procedure. We propose a new method for reconstructing the aortic valve using the autologous pericardium, based on the diameter of the fibrous ring, which can be measured either preoperatively (our preferred option) or intraoperatively.

## Materials and methods

Study design

This study was designed as a retrospective and prospective comparative analysis of two AVNeo techniques: the formula method (FM) and the template method (TM). Out of 64 adult patients who underwent aortic valve neocuspidization (AVNeo) at the Department of Cardiovascular Surgery of I.M. Sechenov First Moscow State Medical University between January 21, 2019 and December 15, 2022, a total of 31 patients who received an isolated procedure were recruited. This selection was restricted to isolated AVNeo in order to effectively compare the two techniques. The patients were divided into two groups according to the technique of AVNeo: FM (17 patients) and TM (14 patients).

The primary endpoint was the occurrence of major adverse valve-related events (MAVRE), including cardiac death, reoperation, and infective endocarditis (IE). Secondary endpoints included the development of significant aortic regurgitation (AR) greater than grade 2, a peak pressure gradient (PPG) greater than 30 mm Hg, an aortic valve area (AVA) less than 1.0 cm², and New York Heart Association (NYHA) functional class III or IV status. The primary goal of the study was to evaluate the intraoperative, early postoperative, and mid-term outcomes of the two methods, providing the first direct comparison between the FM and TM for AVNeo.

The study was conducted in compliance with the ethical requirements of the Ministry of Health of the Russian Federation Order No. 922n, dated November 15, 2012, "On the approval of the Procedure for providing medical care to the adult population in the field of 'surgery'." The study was also approved by the Institutional Review Board of I.M. Sechenov First Moscow State Medical University. Patients were informed of the procedure and were required to sign informed consent for surgery. Individual written consent to publish their study data was also obtained.

Data collection

The surgery was conducted with continuous TEE monitoring, and follow-up adhered to the standard protocols for post-cardiac surgery care. Transthoracic echocardiography was performed at discharge, at three months, and annually by the referring cardiologist. A clinic study nurse collected the data, which was stored in a computerized database. Standard demographic, clinical, TEE, and transthoracic echocardiography data were recorded for cardiovascular studies, with symptoms categorized according to the NYHA functional classification.

Statistical analysis

Data were analyzed using the Statistica 8.0 software package (StatSoft Inc., Tulsa, USA). Continuous variables were assessed for normal distribution using the Shapiro-Wilk test and were expressed as mean values (M) ± standard deviation (SD) or as medians (Me) with interquartile ranges (Q25%-Q75%) for non-normally distributed variables. Categorical variables were compared using the Chi-square test with Yates' continuity correction. For comparison of normally distributed numerical data between groups, analysis of variance (ANOVA) was employed, followed by pairwise comparisons using the Student’s t-test for independent samples. Differences between survival rates and freedom from reoperations were analyzed using Kaplan-Meier curves, and the Cox-Mantel criterion was used to compare survival between groups. Statistical significance was set at p < 0.05.

Surgical techniques

Based on morphometric studies of 54 normal cadaveric aortic valves, key universal equations were derived to determine cusp size based on the intercommissural distance (IC). Cusp free margin horizontal length (L1) = 1.04 x (IC) + 6.17; cusp attachment length (L2) = 1.21 x (IC) + 18.9; and cusp height (H) = 0.33 x (IC) + 10.05. Although these formulas are accurate, they are not easy to apply intraoperatively, which is why we developed a simpler formula based on the study results. Our formulas can be applied by surgeons of any experience level to trim neocusps with ease.

**Figure 1:**
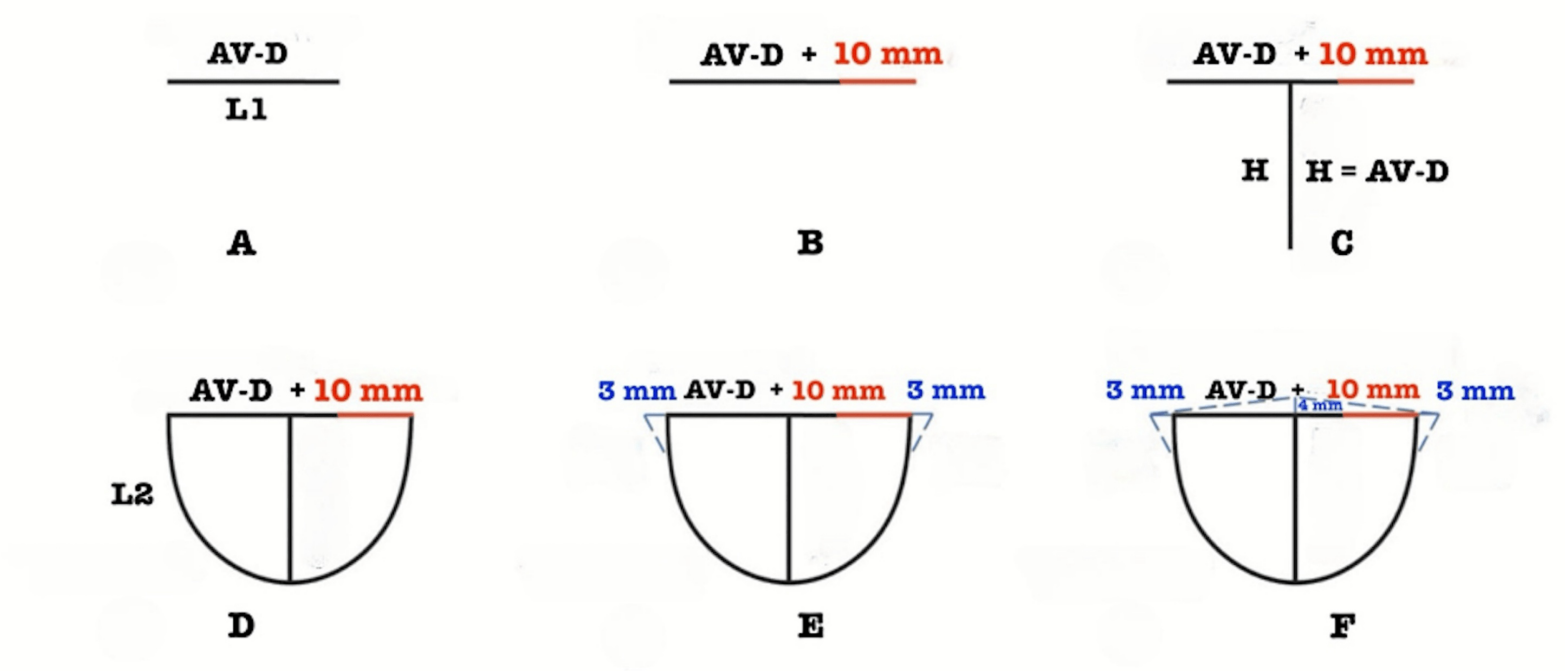
Formula method of trimming neocusps from autologous pericardium. Cusp free margin horizontal length, L1 = AV-D + 10 mm; Cusp height, H = AV-D; L2 parabolically joins L1 and H; Cusp wing = 3mm; AV-D is Diameter of aortic valve fibrous ring

The key innovation in the FM group is that neocusp sizes are calculated intraoperatively based on the diameter of the aortic valve's fibrous ring (AV-D). The dimensions of the aortic valve cusps are represented by IC, H, L1, and L2. Additionally, "wings" and the free margin (roof) are included in the cusps to ensure better coaptation (Figure 1).

Our technique (FM) proposes creating symmetric neocusps using the following simplified formula: L1 = AV-D + 10 mm; H = AV-D (height is measured from the midpoint of L1) (Figures 1A-1C). The L2 is a broad parabola connecting each end of L1 to H (Figure 1D). Next, "wings" are created on both sides of L1 as 3 mm extensions from the junction of L1 and L2. The "wings" are designed for suture placements to secure the free margins to the aortic sinus of Valsalva. Along L2, a line equal to 1/3 of the AV-D (approximately 7-8 mm) is drawn to join the end of the "wings." These newly created segments are connected with a hypotenuse to form a right triangle (Figure 1E). From the midpoint of L1, a 4 mm perpendicular line is drawn in the opposite direction from H. Connecting this point to the ends of L1 forms the free margin (roof) of the neocusp, which improves the coaptation of all valve structures, reducing the likelihood of AR (Figure 1F).

In the TM group, AVNeo templates (JOMDD, Tokyo, Japan) were used to create neocusps After measuring the intercommissural distances of the aortic root, the appropriate templates are used to cut the pericardium into leaflets of a standard size and shape.

After performing a midline sternotomy, the pericardium is carefully excised, typically measuring 7 cm by 8 cm. Any excess fibrofatty tissue is meticulously removed, and the pericardial tissue is immersed in 0.6% glutaraldehyde for 10 minutes for fixation. After this, the tissue is rinsed in three containers with 0.9% normal saline for six minutes each to ensure it is properly prepared for use. An aortotomy is performed 1.5 cm above the right coronary artery, and the diseased valve cusps are removed. Complete decalcification of the annulus is achieved using a surgical rongeur or cavitron ultrasonic surgical aspirator.

**Figure 2:**
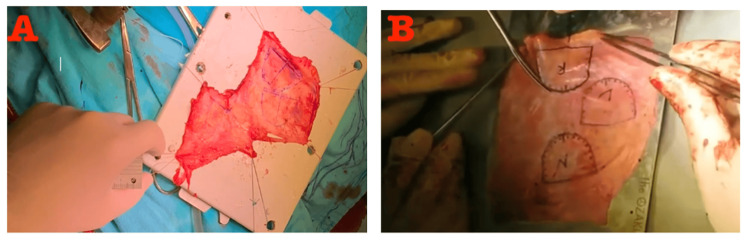
Marking out neocusps on glutaraldehyde-treated autologous pericardium with a pen. (A) Neocusps marking according to the formula method; (B) Neocusps marking according to the template method Zoom in to clearly see the pen markings of the neocusps in the Formula Method in Figure 2A.

Depending on the method of neocusps formation, the treated autologous pericardium is marked using a surgical pen. The cusps are carefully trimmed along the marked lines (Figure 2). The suturing process begins with the right coronary cusp, using 4-0 Prolene sutures, starting at the nadir of each sinus and progressing from the rough to the smooth side of the cusps. After the right coronary cusp is completed, the same process is applied to the other cusps, including the left coronary and non-coronary cusps. The commissures are then anchored to the aortic wall with additional sutures. The remaining non-coronary cusp is sutured using the same technique. Once all three commissures are securely fixed and their heights adjusted for uniformity, the aortotomy is closed. The patient is weaned off cardiopulmonary bypass, and a transesophageal echocardiogram is performed to verify proper valve function, ensuring smooth motion of the leaflets and proper coaptation.

Patients were discharged with a prescription of 100 mg/day aspirin. Anticoagulation therapy was only prescribed when clinically indicated, such as for patients with atrial fibrillation. Other medications were administered as needed. Clinical examinations, including TEE, were conducted before discharge and annually, assessing ejection fraction, AVA, AVR grade, pressure gradients, and pulmonary pressures. Symptoms were classified according to the NYHA functional class.

## Results

Demographics and baseline characteristics

The baseline demographic and clinical characteristics of the patients are presented in Table 1. The mean age of patients in the FM group was younger compared to the TM group (FM: 48.18 ± 18.37 years; TM: 62.71 ± 11.12 years, p = 0.0148). Both groups had a balanced distribution of males and females, and the prevalence of comorbidities such as hypertension and diabetes was similar.

**Table 1:** Baseline demographic and clinical characteristics *p < 0.005 represents a significant difference in comparison between the two groups TM: Template method; FM: formula method

Parameter	FM Group (n = 17)	TM Group (n = 14)	p-value
Age (years)	48.18 ± 18.37	62.71 ± 11.12	0.0148
Sex			
Male (%)	6 (35.3)	6 (42.9)	0.6683
Female (%)	11 (64.7)	8 (57.1)	0.6683
BMI, kg/m^2^	25.94 ± 5.96	26.00 ± 4.89	0.9766
NYHA functional class (average)	1.65 ± 1.06	2.36 ± 0.74	0.0431
NYHA functional class I-II, n (%)	9 (52.9)	7 (50.0)	0.792
NYHA functional class II-IV, n (%)	8 (47.1)	7 (50.0)	0.721
Coronary artery disease, n (%)	0	2 (14.3)	0.108
Hypertension, n (%)	9 (52.9)	13 (92.9)	0.015*
Atrial fibrillation, n (%)	2 (11.8)	1 (7.1)	0.665
Renal failure	2 (11.8)	3 (21.4)	0.467

According to the echocardiography data, the preoperative left ventricular ejection fraction (LVEF) was significantly higher in the group FM compared to the group TM (63.35 ± 4.99% vs. 57.79 ± 8.03%, p = 0.0251). Additionally, the patients in the FM group had a lower preoperative left ventricular mass index (LVMI) (215.49 ± 100.29 g/m² vs. 320.56 ± 157.49 g/m², p = 0.0346), a smaller left atrial volume (45.35 ± 16.84 ml vs. 66.79 ± 24.15 ml, p = 0.007), and a smaller aortic size at the level of the Valsalva sinuses (3.04 ± 0.42 cm vs. 3.43 ± 0.39 cm, p = 0.0133). The FM group also had a lower pulmonary artery pressure (32.76 ± 10.95 mm Hg vs. 40.64 ± 13.37 mm Hg, p = 0.0815). More detailed data on the analyzed preoperative echocardiography parameters are presented in Table 2.

**Table 2:** Preoperative echocardiography parameters *p < 0.005 represents significant difference in comparison between the two groups

Parameter	FM Group (n = 17)	TM Group (n = 14)	p-value
Aortic stenosis (AS), n (%)	7 (41.2)	3 (21.4)	0.242
Aortic regurgitation (AR), n (%)	1 (5.9)	2 (14.3)	0.431
AS + AR, n (%)	9 (52.9)	9 (64.3)	0.525
Left ventricular ejection fraction (%)	63.35 ± 4.99	57.79 ± 8.03	0.025 *
Bicuspid valve	9 (52.9)	4 (28.6)	0.172
Tricuspid valve	8 (47.1)	10 (71.4)	0.012*
Annulus diameter, mm	2.18 ± 0.29	2.22 ± 0.19	0.622
Aortic valve area, cm^2^	0.84 ± 0.24	0.76 ± 0.31	0.452
Aortic size at level of Sinus of Valsalva, cm	3.04 ± 0.42	3.43 ± 0.39	0.013*
Left atrial volume, ml	45.35 ± 16.84	66.79 ± 24.15	0.007*
Peak pressure gradient (PPG), mmHg	79.9 ± 27.6	87.3 ± 33.3	0.504
Mean pressure gradient (PPG), mmHg	46.5 ± 17.5	50.1 ± 21.5	0.609
Peak flow velocity, mm/s	415.8 ± 95.2	450.5 ± 104.9	0.342
End diastolic volume, ml	93. 00 ± 43. 03	107.71 ± 35.78	0.315
End systolic volume, ml	34.12 ± 17.29	46.79 ± 23.78	0.096
End diastolic size, cm	4.72 ± 1.06	5.26 ± 1.31	0.2136
End systolic size, cm	3.51 ± 0.51	3.32 ± 0.56	0.376
Interventricular septal thickness, cm	1.22 ± 0.22	1.37 ± 0.24	0.068
Posterior wall thickness, cm	0.95 ± 0.22	1.09 ± 0.21	0.067
Left ventricular myocardial mass, kg	215.49 ± 100.29	320.56 ± 157.49	0.032*
Pulmonary arterial pressure, mm Hg	32.76 ± 10.95	40.64 ± 13.37	0.081

Perioperative outcomes

Intraoperative parameters, including cardiopulmonary bypass (CPB) time, myocardial ischemia time, and blood loss, are shown in Table 3. The CPB time was slightly longer in the FM group, but this difference was not statistically significant (FM: 126.8 ± 33.3 min; TM: 118.6 ± 19.1 min, p = 0.497). Similarly, myocardial ischemia times were comparable between the groups. Blood loss was nearly identical between both techniques (FM: 673.53 ± 153.21 ml; TM: 671.4 ± 80.18 ml, p = 0.963). The mean duration of ICU stay was similar between the groups (FM: 1.6 days; TM: 1.2 days, p = 0.112), as was the total hospital stay (FM: 13.2 ± 2.8 days; TM: 13.4 ± 3.0 days, p = 0.611). There were no significant differences in major complications, such as stroke, infection, or bleeding, during the early postoperative period. Neither group experienced any conversions to mechanical or biological prostheses during surgery. No other perioperative complications were recorded.

**Table 3:** Perioperative parameters CPB: Cardiopulmonary bypass; ICU: Intensive care unit

Parameter	FM Group (n =17)	TM Group (n = 14)	p-value
CPB time, mins	126.8 ± 33.3	118.6 ± 19.1	0.497
Aortic cross-clamp time, mins	93.12 ± 18.85	85.14 ± 18.16	0.243
Blood loss, ml	673.53 ± 153.21	671.4 ± 80.18	0.963
Total operation time, ml	335.59 ± 65.17	319.5 ± 33.42	0.410
Transient cardiac arrhythmias, n (%)	2 (11.8)	4 (28.6)	0.239
ICU stay, days	1.6	1.2	0.112
Hospital stay, days	13.2 ± 2.8	13.4 ± 3.0	0.611
In-hospital mortality	2	0	0.185
Aortic regurgitation ≥ grade 2, n (%)	0	0	1.0
Left ventricular ejection fraction, %	63.13 ± 4.51	59.14 ± 8.14	0.103
Annulus diameter, mm	14.69 ± 5.56	15.07 ± 4.55	0.839
Peak pressure gradient (PPG), mmHg	14.69 ± 5.56	15.07 ± 4.55	0.839
Mean pressure gradient (PPG), mmHg	7.06 ± 2.72	7.07 ± 2.23	0.992
Peak flow velocity, mm/s	185.25 ± 28.17	189.5 ± 29.4	0.684

Mid-term outcomes

The average follow-up time after surgery in the FM group was 11.1 ± 7.5 months, while in the TM group, it was 15.8 ± 10.0 months (p = 0.513). Mid-term outcomes showed no statistically significant differences in valve function or hemodynamic performance between the two groups (Table 4). Peak and mean pressure gradients across the valve were comparable (p = 0.219 and p = 0.155 respectively). Both groups also demonstrated similar results for peak flow velocity and incidence of significant aortic regurgitation (AR ≥ Grade 2). Left ventricular remodeling was significant in both groups, with substantial reductions in left ventricular mass observed after surgery; however, there was a significant difference in the postoperative left ventricular mass reduction between the groups.

**Table 4:** Mid-term hemodynamic outcomes

Parameter	FM Group (n = 17)	TM Group (n = 14)	p-value
Peak pressure gradient (PPG), mmHg	14.1 ± 4.3	18.4 ± 12.0	0.219
Mean pressure gradient (MPG), mmHg	6.8 ± 1.7	9.8 ± 7.6	0.155
Peak flow velocity, cm/s	182.7 ± 25.7	208.4 ± 59.7	0.151
Aortic valve area, cm^2^	2.43 ± 0.3	2.4 ± 0.2	0.890
Aortic regurgitation ≥ grade 2, n (%)	1 (5.9)	0	0.309
Left ventricular ejection fraction, %	63.64 ± 3.5	60.64 ± 6.4	0.136
Reoperations, n (%)	0	0	1.0
Cumulative mortality, n (%)	3 (17.6)	0	0.099
Aortic valve area, cm^2^	2.43 ± 0.3	2.4 ± 0.2	0.890
End diastolic volume, ml	85.43 ± 25.7	97.44 ± 29.0	0.793
End systolic volume, ml	31.27 ± 10.8	36.29 ± 19.2	0.402
End diastolic size, cm	4.30 ± 0.5	4.29 ± 0.7	0.947
End systolic size, cm	2.82 ± 0.4	2.94 ± 0.6	0.540
Interventricular septal thickness, cm	1.2 ± 0.2	1.2 ± 0.2	0.948
Posterior wall thickness, cm	1.0 ± 0.2	1.0 ± 0.2	0.972
Left ventricular myocardial mass, kg	180.0 ± 54.3	204.8 ± 105.0	0.439

There were no statistically significant differences in key hemodynamic outcomes between the groups (p > 0.05). However, while not statistically significant, the FM group demonstrated a trend toward better overall hemodynamic performance in the mid-term follow-up. Overall, both techniques were shown to be effective, with similar intraoperative, postoperative, and mid-term outcomes.

## Discussion

Despite the growing popularity of AVNeo and the existence of its several variations, the method developed by S. Ozaki remains the most widely adopted [9]. It is worth noting that this method involves asymmetry of the neocusps, which may initially seem logical, but in the long term, it proves inefficient due to high levels of regurgitation [19,21,22] and valve dysfunction [13,20]. The lack of a universal approach to AVNeo, along with knowledge of the hemodynamics of aortic root structures, has led to the development of our innovative FM for calculating neocusp dimensions that are hemodynamically comparable to the commonly accepted template-based method.

This study represents a comparison between the FM, a novel template-free approach to AVNeo, and the established TM, which utilizes specialized templates for leaflet trimming. The primary outcomes, including intraoperative parameters, early postoperative recovery, and mid-term hemodynamic performance, indicate that both techniques are effective for AVNeo.

Postoperatively, both groups showed excellent early recovery outcomes, with no significant differences in ICU stay, hospital stay, or the incidence of early complications. Importantly, mid-term outcomes showed that both techniques produced comparable valve function and hemodynamic performance, as reflected by similar peak pressure gradients, mean gradients, and peak flow velocities across the valve. Additionally, patients in both the FM group and TM group experienced significant reverse left ventricular remodeling, underscoring the effectiveness of both methods in promoting cardiac recovery after AVNeo.

The primary advantage of the FM is its cost-effectiveness. By forgoing the need for specialized templates and sizers, the FM reduces the overall cost of AVNeo while maintaining comparable clinical outcomes to the TM. This is particularly relevant in resource-limited settings, where access to expensive surgical equipment may be limited. Additionally, the flexibility of the FM, which tailors valve leaflet sizes based on preoperative and/or intraoperative measurements of the patient's aortic anatomy, ensures a personalized approach that can adapt to individual anatomical variations. This offers the potential for broader application across different patient populations and varying levels of surgical expertise.

Many authors confirm that AVNeo by the Ozaki method (TM) typically replicates the anatomy of a normal aortic valve and therefore provides a larger effective orifice area and lower postoperative gradients [23-25]. Ozaki et al. reported an average peak gradient of 15.2±6.3 mmHg eight years after surgery [17]. Krane and colleagues noted an average pressure gradient of 8.9±3.8 mmHg and an effective orifice area of 2.1±0.7 cm² at discharge, which remained stable during the first postoperative year [24]. According to the results of a meta-analysis by Benedetto et al., the peak and mean transvalvular gradients were 16.0±3.7 and 9.0±2.2 mmHg, respectively, after 12.5 months [23]. In a meta-analysis that included 1,891 patients, the peak pressure gradient was 15.7±7.4 mmHg over a follow-up period of 38.1±23.8 months [26].

In this study, mid-term hemodynamic parameters such as peak pressure gradient, mean pressure gradient, and peak flow velocity in the FM group were comparable to those for the TM group (14.1 ± 4.3 mmHg, 6.8 ± 1.7 mmHg, and 182.7 ± 25.7 cm/s versus 18.4 ± 12.0 mmHg, 9.8 ± 7.6 mmHg, and 208.4 ± 59.7 cm/s; p = 0.219, p = 0.155, p = 0.151 respectively). The immediate postoperative hemodynamic parameters were likewise comparable between the two groups. The effective orifice area was 2.43 ± 0.3 cm² in the FM group versus 2.40 ± 0.2 cm²/m² in the TM group (p = 0.890). These results are comparable to those of the previous studies [17,23,24,26].

Undoubtedly, the operative duration of AVNeo is longer than aortic valve replacement (AVR) with prosthetic valves [27]. However, according to a meta-analysis of 22 studies with 1,891 patients, the average aortic clamp and CPB times were 106.8±24.8 minutes and 135.2±35.1 minutes, respectively [26], which are comparable to the times in this study (93.12 ± 18.85 minutes and 126.8 ± 33.3 minutes in the FM group vs 85.14 ± 18.16 and 118.6 ± 19.1 minutes in the TM group; p = 0.243 and p = 0.497, respectively). Ozaki et al. reported freedom from death, cumulative rates of reoperations, and recurrence of AR ≥ grade 2 after an average follow-up period of 53.7±28.2 months in 85.9%, 4.2%, and 7.3% of cases, respectively [28]. In this study, one case of recurrent AR ≥ grade 2 and three deaths were recorded in the FM group after discharge, whereas no such complications were recorded in the TM group at midterm follow-up (11.1 ± 7.5 months in the FM group and 15.8 ± 10.0 months in the TM group; p = 0.513).

Both immediate and mid-term results in the two groups are comparable, making FM a viable alternative to TM. Additionally, the results of AVNeo by both methods are comparable to those of a native valve in terms of valve parameters, suggesting that both methods are more anatomically correlated to the native aortic valve than AVR with a prosthetic valve. AVNeo itself provides relatively high resistance to infective endocarditis pathogens, making it a potential treatment option for patients with aortic valve infective endocarditis; it should also be considered for patients with systemic inflammatory diseases when other correction methods are unavailable or undesirable [29]. In this study and others, AVNeo has been shown to allow for an optimal effective orifice area, which, combined with low gradients, contributes to significant myocardial reverse remodeling, thereby not only improving heart function but also reducing complications [30].

Although data from other centers are limited, recent studies with smaller cohorts (30 and 52 patients) have also yielded encouraging midterm results with low reoperation rates and low echocardiographic gradients [24,30]. Our results are consistent with these studies. In addition, no case requiring permanent pacemaker implantation was recorded in this study. Ozaki et al. [28] and Krane et al. [24] reported a lower incidence of conduction issues. Long-term results may be further improved by mitigating degenerative factors; the excellent hemodynamic outcomes and the use of autologous material could enhance durability in AVNeo. Autologous tissue has superior biocompatibility compared to xenogeneic tissues, which may contribute to better long-term results. However, the use of glutaraldehyde for crosslinking poses risks, as it is cytotoxic, proinflammatory, and contributes to graft calcification [31,32].

Despite the promising results, the FM is not without challenges. One potential limitation is that the absence of standardized templates could introduce variability in the trimming of neocusps, especially among less experienced surgeons. The success of the FM relies on precise intraoperative measurements and the surgeon’s ability to apply the mathematical formula to trim the valve leaflets accurately. This could pose a learning curve for surgeons who are more accustomed to using templates to ensure uniformity in leaflet formation.

Furthermore, while the early and mid-term results of this study are encouraging, long-term data on the durability of the valve leaflets created using the FM are still limited. Future studies with a larger patient cohort, concomitant cardiac procedures, and longer follow-up periods will be necessary to confirm whether the hemodynamic benefits of the FM are maintained over time and to assess the incidence of long-term complications such as calcification, valve thrombosis, or structural valve deterioration. Additionally, the application of the FM to different patient populations, such as pediatric patients or those with complex aortic pathologies, warrants further investigation. We plan to continue long-term monitoring of our patients. Additionally, our study was non-randomized, conducted at a single center, and involved a relatively small sample size.

## Conclusions

In conclusion, the FM offers a significant advancement in aortic valve neocuspidization, complementing the strengths of the template method while addressing some of its limitations. The potential for broader adoption of the FM, particularly in resource-constrained environments, makes it a valuable tool in the global fight against aortic valve disease. With continued research, refinement, and clinical validation, the FM could play a transformative role in the future of cardiac surgery, providing high-quality care to a wider range of patients and improving global cardiovascular outcomes.
